# Integrative multi-omics analysis depicts the methylome and hydroxymethylome in recurrent bladder cancers and identifies biomarkers for predicting PD-L1 expression

**DOI:** 10.1186/s40364-023-00488-3

**Published:** 2023-05-03

**Authors:** Zhen-Duo Shi, Xiao-Xiao Han, Zi-Jian Song, Yang Dong, Kun Pang, Xin-Lei Wang, Xin-Yu Liu, Hao Lu, Guang-Zhi Xu, Lin Hao, Bing-Zheng Dong, Qing Liang, Xiao-Ke Wu, Cong-Hui Han

**Affiliations:** 1 Department of Urology, Xuzhou Clinical School of Xuzhou Medical University, Jiangsu, China; 2 Department of Urology, Xuzhou Central Hospital, Xuzhou, Jiangsu China; 3 School of Life Sciences, Jiangsu Normal University, Jiangsu, China; 4 Department of Urology, Heilongjiang Provincial Hospital, 82 Zhongshan Road, Xiangfang District, Harbin City, Heilongjiang Province China; 5 Clinical and Translational Research Center of Shanghai First Maternity and Infant Hospital, Shanghai Key Laboratory of Signaling and Disease Research, Frontier Science Center for Stem Cell Research, School of Life Sciences and Technology, Tongji University, Shanghai, China; 6 Department of Urology, Ren Ji Hospital, Shanghai Jiao Tong University School of Medicine, Shanghai, China; 7 Department of Reproductive Medicine, Heilongjiang Provincial Hospital, 82 Zhongshan Road, Xiangfang DistrictHeilongjiang Province, Harbin City, China; 8 Department of Gynaecology and Obstetrics, Heilongjiang Provincial Clinical Research Centre for Ovary Diseases, First Affiliated Hospital, Heilongjiang University of Chineses Medicine, 26 Heping Road, Xiangfang District, Harbin, Heilongjiang China

## Abstract

**Background:**

Urinary bladder cancer (UBC) is a common malignancy of the urinary tract; however, the mechanism underlying its high recurrence and responses to immunotherapy remains unclear, making clinical outcome predictions difficult. Epigenetic alterations, especially DNA methylation, play important roles in bladder cancer development and are increasingly being investigated as biomarkers for diagnostic or prognostic predictions. However, little is known about hydroxymethylation since previous studies based on bisulfite-sequencing approaches could not differentiate between 5mC and 5hmC signals, resulting in entangled methylation results.

**Methods:**

Tissue samples of bladder cancer patients who underwent laparoscopic radical cystectomy (LRC), partial cystectomy (PC), or transurethral resection of bladder tumor (TURBT) were collected. We utilized a multi-omics approach to analyze both primary and recurrent bladder cancer samples. By integrating various techniques including RNA sequencing, oxidative reduced-representation bisulfite sequencing (oxRRBS), reduced-representation bisulfite sequencing (RRBS), and whole exome sequencing, a comprehensive analysis of the genome, transcriptome, methylome, and hydroxymethylome landscape of these cancers was possible.

**Results:**

By whole exome sequencing, we identified driver mutations involved in the development of UBC, including those in *FGFR3*, *KDMTA,* and *KDMT2C*. However, few of these driver mutations were associated with the down-regulation of programmed death-ligand 1 (*PD-L1*) or recurrence in UBC. By integrating RRBS and oxRRBS data, we identified fatty acid oxidation-related genes significantly enriched in 5hmC-associated transcription alterations in recurrent bladder cancers. We also observed a series of 5mC hypo differentially methylated regions (DMRs) in the gene body of *NFATC1*, which is highly involved in T-cell immune responses in bladder cancer samples with high expression of *PD-L1*. Since 5mC and 5hmC alternations are globally anti-correlated, RRBS-seq-based markers that combine the 5mC and 5hmC signals, attenuate cancer-related signals, and therefore, are not optimal as clinical biomarkers.

**Conclusions:**

By multi-omics profiling of UBC samples, we showed that epigenetic alternations are more involved compared to genetic mutations in the *PD-L1* regulation and recurrence of UBC. As proof of principle, we demonstrated that the combined measurement of 5mC and 5hmC levels by the bisulfite-based method compromises the prediction accuracy of epigenetic biomarkers.

**Supplementary Information:**

The online version contains supplementary material available at 10.1186/s40364-023-00488-3.

## Introduction

Urinary bladder cancer (UBC) is a common malignancy of the urinary tract and one of the major causes of cancer-related deaths worldwide [1]. Approximately 75% of UBCs are non-muscle-invasive [2, 3]. Although early-stage UBCs are treatable by transurethral resection of bladder tumors, these are characterized by frequent recurrence rates as high as 60–80% [4]. Guidelines recommend lifelong surveillance for recurrent UBC through cystoscopy [5]. However, cystoscopy is invasive, painful, and costly. Furthermore, its unsatisfactory sensitivity may lead to missed cases of bladder malignancies reaching up to 10–40% [6]. Urine cytology is a complementary tool but is hampered by suboptimal sensitivity, especially in low-grade UBCs [7, 8]. Therefore, there is an urgent need for developing accurate, efficient, and preferably non-invasive screening and diagnostic methods for UBC.

DNA methylation and hydroxymethylation are epigenetic mechanisms that covalently affect DNA and can be exploited as DNA biomarkers. DNA methylation is conventionally acknowledged as a silencing epigenetic marker and is promoted by DNA methyltransferases (DNMTs), generating 5-methylcytosine (5-mC or 5mC) [9]. In contrast, hydroxymethylation involves an oxidative process that converts 5-mC to 5-hydroxymethylcytosine (5-hmC or 5hmC). This reaction is promoted by a family of dioxygenases, namely ten-eleven translocation proteins (TETs). Through hydroxymethylation, TETs can help maintain an unmethylated state, thus playing the role of an activator of the intracellular transcription [10]. 5hmC tends to exist in promoter regions with dual histone markers, including H3K4me3 and H3K27me3 for expressional activation and repression, respectively. Thus, 5hmC may be involved in regulating gene expression by recruiting activators or repressors. Genomic hypermethylation, which may result from a disturbed balance between methylation and hydroxymethylation, can lead to aberrant silencing of tumor suppressors and DNA repair enzymes, further resulting in accelerated carcinogenesis [11]. 5hmC modification levels decrease in cancers, including bladder cancer. However, few studies have investigated 5hmC levels in bladder cancers at base resolution.

Aberrant DNA hydroxymethylation is a hallmark of various malignancies [12]. Forloni et al. [13] demonstrated that TET was transcriptionally downregulated by oncogenic epidermal growth factor receptors in lung cancers, thus silencing diverse tumor suppressors. The same was found in colorectal cancer by Neri et al. [14]. TET normally inhibits tumor growth by repressing the WNT signaling pathway through the demethylation of promoters of WNT inhibitors. In colon cancer tissues, downregulation of TET lead to insufficient DNA hydroxymethylation of WNT inhibitors and accelerates colon carcinogenesis. Other malignancies potentially affected by aberrant DNA hydroxymethylation include prostate cancer, breast cancer, ovarian cancer, skin cancer, and several hematopoietic malignancies (e.g., acute myeloid leukemia, chronic myelomonocytic leukemia, and T-cell lymphomas) [14–16]. Notably, aberrant DNA hydroxymethylation is also considered one of the earliest events in urothelial carcinomas [16, 17]. A global loss of 5-hmC compared to controls or adjacent tissues has been observed in both UBC and UBC cell lines [15]. Therefore, similar to the conventional DNA methylation level measured based on bisulfite treatment, the hydroxymethylation level may also be a promising biomarker for UBC detection, especially in the repetitive surveillance of recurrent UBC.

In the present study, we used a multi-omics approach to investigate the possible use of 5mC and 5hmC levels (base-resolution) as urinary cancer biomarkers for associating crucial clinical outcomes and investigating their diagnostic values in UBC.

## Materials and Methods

### Clinical samples

The study was performed at the Urology Surgery department, Xuzhou Central Hospital, Xuzhou 221,009, PR China. The subjects were recruited from 2020.09 to 2021.02. Bladder cancer patients who underwent laparoscopic radical cystectomy (LRC), partial cystectomy (PC), or transurethral resection of bladder tumor (TURBT) were selected and bladder tumor tissues and paracancerous tissues were collected. The tissues were cleaned with sterile normal saline immediately after the surgery and stored at -80* °C* until further use. A total of 44 bladder tissue specimens were collected from 44 patients, including 44 bladder cancer tissues and 10 paracancerous tissues.

The following exclusion criteria were applied: patients with other types of tumors; those with a postoperative pathological diagnosis of non-bladder cancer; individuals with co-existing serious conditions such as cardiovascular diseases that could result in an expected survival of fewer than 6 months, and those with incomplete clinical data.

### DNA extraction and whole exome sequencing (WES) library construction

Genomic DNA was extracted from bladder cancer tissues. DNA quantification and integrity were determined by the Nanodrop spectrophotometer (Thermo Fisher Scientific, Inc., Wilmington, DE) and 1% agarose gel electrophoresis, respectively. Genomic DNA samples were captured using the Agilent SureSelect Human All Exon v6 library following the manufacturer’s protocol (Agilent Technologies, USA). Briefly, approximately 130 μl (3 μg) genomic DNA was sheared to 150–220 bp small fragments using a sonicator (Covaris, Inc., Woburn, MA). The sheared DNA was purified and treated with reagents in the kit according to the specified protocol. Adapters from Agilent were ligated onto the polished ends and the libraries were amplified by polymerase chain reaction (PCR). The amplified libraries were hybridized with the custom probes. The DNA fragments bound with the probes were washed and eluted with the buffer provided in the kit. Then, these libraries were sequenced on the Illumina sequencing platform (HiSeq X-10, Illumina, Inc., San Diego, CA), and 150 bp paired-end reads were generated. WES and analyses were conducted by OE Biotech Co., Ltd. (Shanghai, China).

### RNA-seq library construction

The RNA-seq libraries were constructed by E-GENE Co. Ltd. Briefly, the total RNA from each sample was extracted using the Invitrogen TRIzol® Reagent and then treated with RNase-free DNase I for 30 min following the manufacturer’s protocols. The poly(A) containing mRNA was purified using Oligo(dT) Beads from 1 μg total RNA. The captured mRNA was first fragmented into 100–200nt using divalent cations at elevated temperatures. The fragmented mRNA was reverse transcribed with SuperScript II and then converted to double-stranded cDNA using RNaseH and DNA Pol I by random priming. After purification, the double-stranded cDNA was subjected to blunt-ending, dA addition to 3’-end, and adapter ligation. Finally, PCR was conducted to enrich the adapter-ligated cDNA and libraries were analyzed by Agilent Bioanalyzer 2100 and quantified by qPCR before sequencing on the Illumina sequencing platform.

### oxRRBS- and RRBS-seq library construction

Briefly, 2 µg genomic DNA was digested using MspI enzyme for 16 h at 37* °C*. After digestion, libraries were constructed following the Illumina Pair-End protocol with some modifications. Specifically, purified digested DNA was subsequently treated with a mix of T4 DNA polymerase, Klenow Fragment, and T4 polynucleotide kinase to repair, blunt, and phosphorylate ends. Subsequently, sequencing control DNA supplied by the TrueMethyl Seq Kit (CEGX) was mixed with the blunt DNA. The mixture of DNA fragments was subsequently 3’ adenylated using the Klenow Fragment (3’-5’ exo-) and followed by ligation to adaptors synthesized with 5’-methylcytosine instead of cytosine using T4 DNA Ligase. The DNA was purified using QIAquick PCR purification kit (Qiagen) after the reaction was performed in each step.

Before the oxidation reaction, all products were purified using Magnetic Beads Binding Solution 1 supplied in the TrueMethyl Seq Kit (CEGX) following the manufacturer’s instructions. After purification, an oxidation reaction was conducted in a reaction volume of 25 µl with 1 µl of the Oxidant Solution (CEGX) for each oxRRBS library, and the RRBS library sample was added in 1 μl of ultrapure water instead of the Oxidant, as a control. Both libraries were subjected to 40 °C, 30 min treatment in a thermocycler with the lid preheated to 57 °C. Next, the reaction mixture was centrifuged at 14,000 xg for 10 min and the supernatant was transferred into a new 0.2 ml PCR tube for the bisulfite treatment. Bisulfite conversion treatment was performed using a TrueMethyl Seq Kit (CEGX) according to the manufacturer’s instructions. The final oxRRBS and RRBS libraries were generated by PCR amplification using adapter-compatible barcode primers, quantified by an Agilent 2100 Bioanalyzer (Agilent Technologies) and real-time PCR assay, and sequenced on the Illumina Hiseq platform.

### 5mC/5hmC-specific qPCR

Clinical samples embedded in 25 wax blocks were cut into 10 µm-thick sections and sample nucleic acids were extracted using the QIAamp DNA FFPE Tissue Kit (Qiagen, USA). BisulPlus™ Loci 5mc & 5hmC Detection PCR Kit (Epigentek, USA) was used to modify the genomic DNA with bisulfite according to the manufacturer's instructions. Bisulfite conversion of genomic DNA results in unmethylated cytosine being converted to uracil while methylated cytosines remain unchanged. The bisulfite-modified DNA was further treated with specific APOBEC deaminase, which converts 5mC to T, thus distinguishing it from 5hmC. Using online the CpG Island software, the EZHIP, ALKBH5, and TUBG1 genes (http://emboss.bioinformatics.nl/cgi-bin/emboss/cpgplot) were analyzed. The sequences of the more dense CpG dinucleotide region were selected to design the 5mC and 5hmC primers using the Premier 5.0 software (Primer, Canada). The reaction system was prepared according to the manufacturer's instructions using the amplification reagents in the BisulPlus™ Loci 5mc & 5hmC Detection PCR Kit (Epigentek, USA). The PCR cycling conditions were as follows: 60 s at 95 °C, followed by 45 cycles of 15 s at 95 °C and 30 s at 60 °C, and a final cycle of 15 s at 95 °C, 60 s at 60 °C, 30 s at 95 °C and 15 s at 60 °C. The sequences of PCR primers were as follows: EZHIP (5hmC): PCR forward CCGGTTCTTCCGCTGCAGCCGC and PCR reverse CGTCAGCACAGCAGCTATGATTGGCAGCCC; ALKBH5 (5hmC): PCR forward GGCGGCGCGGCGTGAAGACAG and PCR reverse GCAGACAGGAACCGCTTGCCGTCCG; TUBG1 (5hmC): PCR forward GGGATCTCGCTGTGGGATCCTGGACTCCA and PCR reverse CCCTCGGGGTTGGGCAAGTGGACACTG; EZHIP (5mC): PCR forward CCGGTTTTTTTGTTGTAGTTGT and PCR reverse CATCAACACAACAACTATAATTAACAAGCCC; ALKBH5 (5mC): PCR forward GGTGGTGTGGTGTGAAGATAG and PCR reverse ACAAACAAAAACCACTTACCATCCA; TUBG1 (5mC): PCR forward GGGATTTCGTTGTGGGATTTTGGATTTTA and PCR reverse CCCTCAAAATTAAACAAATAAACACTA.

### Measuring serum HSV IgG levels

The venous blood of patients was collected following uniform standards and sent to the laboratory department of Xuzhou Central Hospital for determination. HSV1/2 IgG expression levels were measured on an AutoLumo A6000 instrument (AutoBio, China) using a herpes simplex virus type 1/2 antibody kit (AutoBio, China) following the manufacturer's instructions.

### Mutation data analysis

We obtained the Fastq data and aligned them to the hg38 genome using the Burrows-Wheeler Aligner. The resulting SAM files were sorted and indexed using Samtools. To analyze the mutation data, we applied GATK (version 4.2.5) best practice (Mutect2), as described in [18]. The resulting VCF files were subjected to filtering and were annotated using Functator ((FUNCtional annOTATOR)). The mutation results were recorded in MAF files. We used Maftools for analyzing the tumor mutations and generating oncoplots. To compare driver mutation frequency, we referred to The Cancer Genome Atlas (TCGA) datasets.

### RNA-seq data analysis

Trimmomatic (v0.39) was used to remove sequences with low-quality and adapter sequences from raw sequence files [19]. Trimmed fastq files were mapped to the hg38 genome reference by using STAR (v2.7.0a) [20]. Alignments were then cleaned using the view function of samtools (v1.9) [21] with the parameter -F set to 268. The read counts mapped to each gene were calculated by Htseq-count (v2.0.2) [22]. Finally, DESeq2 (v1.36.0) [23] was used to identify the differentially expressed genes (DEGs). Significant DEGs were defined by an adjusted P-value (Benjamini-Hochberg) less than 0.05 and an absolute value of log2FoldChange more than 1. Pathway enrichment and Gene Ontology analysis were performed by using Enrichr.

### oxRRBS-seq/RRBS-seq data analysis

TrimGalore (v0.6.5) was used to remove the sequence with low-quality, adapter sequences, and 5 bp from the 5’ end of read R2. The trimmed reads were then aligned to the hg38 human reference genome using BSMAP (v2.90) [24]. The view function of Samtools was used to filter low-quality alignments from the mapped bam file. The methratio.py provided by BSMAP was used for calculating the CpG methylation level in the cleaned bam file. The 5hmC methylation level of each CpG site was calculated by subtracting the 5mC (oxRRBS-seq methylation level) level from the RRBS-seq methylation level. The CpG 5mC or 5hmC ratio was finally processed by metilene (v0.2–8) [25] to call de-novo 5mC or 5hmC differentially methylated regions (DMRs). The significant 5mC DMRs were defined by the absolute value of methylation difference more than 0.2 and adjusted P-value (Benjamini-Hochberg) less than 0.05. The significant 5hmC DMRs were defined by the absolute value of methylation difference more than 0.1 and adjusted P-value (Benjamini-Hochberg) less than 0.05. Pathway enrichment and Gene Ontology analysis were performed by using Enrichr [26].

## Results

### Genomic and transcriptomic profiling of the bladder cancer cohort

We examined the mutations (SNVs, indels) in our UBC samples with different statuses and grades (blue: recurrent, high-grade; red: primary, low-grade) (Figure S1A). The median number of variants per sample was 283.5, and most of these were missense variants. The top ten genes with mutations were *MUC16*, *TTN*, *HRNR*, *PRAG1*, *FAM8A1*, *LNP1*, *CBX3*, *KCNN2*, *DHDH*, and *TMPRSS13* (Figure S1B). All tumors were identified as microsatellite-stable by genome-wide microsatellite analysis. The RTK-RAS, NOTCH, and WNT cascades [27] were identified as the most significantly altered pathways in the enrichment analysis of the mutation datasets (Figure S1C). We next examined the mutation frequency of the known UBC driver mutations and determined that most allele frequencies ranged from 0.2 to 0.8 (Figure S1D, S1E). The mutation frequencies of these driver genes in our cohort were also comparable to those in the known TCGA datasets.

We further performed RNA-seq on 10 bladder paracancer tissues and 40 bladder cancer tissue samples to investigate transcriptomic alterations during bladder cancer oncogenesis. The RNA-seq data were processed using the STAR mapping tool. Using DESeq2, we identified 340 significantly upregulated and 458 downregulated genes with an absolute value of log2FoldChange of more than 1 and adjusted P-value less than 0.05 (Figure S2A). The upregulated genes were the most enriched in the herpes simplex virus 1 (HSV1) infection pathway, suggesting the presence of HSV1 infection in bladder cancer samples. Consistent with previous studies reporting that dysregulation of coagulation is associated with HSV infection, downregulated genes were enriched in the complement and coagulation cascade pathways. The downregulated genes were also enriched in common cancer pathways, such as the PI3K-Akt and MAPK signaling transduction. We also observed dysregulation of genes related to focal adhesion and ECM-receptor interaction, which potentially promotes tumor cell proliferation and mobility (Figure S2B). Overall, herein, we presented a high-quality mutation and RNA-seq dataset for the UBC cohort.

### Depicting the 5mC and 5hmC landscape alternation in bladder cancer through oxRRBS-seq and RRBS-seq

**Fig. 1.**
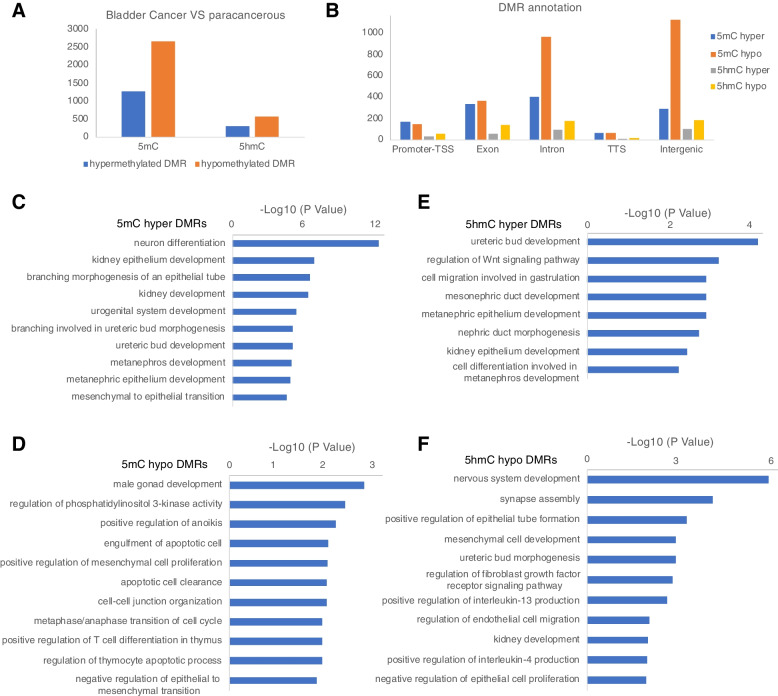
5mC and 5hmC profiling of bladder cancer samples at single-base resolution. **A** The number of significant 5mC and 5hmC differentially methylated regions (DMRs) identified in bladder cancer samples. **B** The genomic annotations of 5mC and 5hmC DMRs. Pathway enrichment for 5mC hyper (**C**) and hypo DMRs (**D**) in bladder cancer. Pathway enrichment for 5hmC hyper (**E**) and hypo DMRs (**F**) in bladder cancer

The role of DNA methylation in bladder cancer oncogenesis has been extensively studied. However, most studies are based on the 450 K methylation array, RRBS, or WGBS, which cannot distinguish 5mC from 5hmC. oxRRBS was developed by Booth et al. [28] to convert 5hmC into 5fC while keeping 5mC unconverted; therefore, it can profile the 5mC status on a genome-wide scale at base resolution. To illustrate the role of 5mC in bladder cancer development, we performed oxRRBS for 10 paracancerous and 44 cancerous bladder tissue samples. Using Metilene, we identified 1266 significantly hypermethylated 5mC DMRs and 2666 hypomethylated 5mC DMRs (Fig. 1A). The DMRs had a minimal mean methylation change of 0.2 with an adjusted P-value of less than 0.05.

Although bladder cancer samples had increased hypomethylated DMRs, a similar number of hyper- and hypo-DMRs were annotated to the promoter, exon, and TTS regions. In contrast, more hypo-DMRs were annotated in the intron and intergenic regions (Fig. 1B). This finding is consistent with previous studies suggesting that hypermethylation occurs in promoter regions, whereas hypomethylation occurs in the gene body and intergenic regions. Hypermethylated genes are highly enriched in the development of the urogenital system and branching involved in ureteric bud morphogenesis, suggesting that bladder cancer progression is accompanied by the disruption of the morphology of the urogenital system. These genes are also enriched in kidney development, such as kidney epithelium and metanephros development, consistent with chronic kidney disease being common in older patients with bladder cancer (Fig. 1C).

Hypo-DMRs were mainly enriched in positive regulation of anoikis, engulfment of apoptotic cells, and apoptotic cell clearance. Additionally, hypo-DMRs were enriched in PI3K pathway activity and cell cycle regulation, which may contribute to the increased proliferative ability of bladder cancer cells. Notably, hypo-DMRs were the most enriched in male gonad development. Given that bladder cancer prevalence is three to four times higher in men, it will be interesting to further investigate whether DNA methylation is involved in the difference in bladder cancer incidence between sexes (Fig. 1D).

Next, we performed RRBS and oxRRBS on 10 bladder paracancer tissue and 44 bladder cancer tissue samples. The 5hmC level was calculated by subtracting the oxRRBS methylation level from RRBS, which profiles both 5hmC and 5mC simultaneously. By comparing bladder cancer tissues with paracancerous tissues, we identified 287 hyper-5hmC DMRs and 534 hypo-5hmC DMRs. The 5hmC DMRs had a minimal mean methylation change of 0.1 and an adjusted P-value of less than 0.05. In contrast to 5mC DMRs, we observed a comparable number of 5hmC hyper-DMRs and hypo-DMRs in the exon, intergenic, intron, and TTS regions but approximately five times more 5hmC hypo-DMRs in promoter regions than 5hmC hyper-DMRs (Fig. 1A and B).

Both 5hmC hyper- and hypo-DMRs were enriched in pathways related to the urinary system, such as ureteric bud development and kidney development, indicating that 5hmC alterations are likely involved in disrupting the urinary system during bladder cancer development. Additionally, 5hmC hypo-DMRs were enriched in nervous system development, regulation of the FGFR signaling pathway, positive regulation of IL-13 and IL-4 production, mesenchymal cell development, and negative regulation of epithelial cell proliferation suggesting that 5hmC also plays a role in regulating the proliferation, immune escape, and epithelial-to-mesenchymal transition (EMT) of bladder cancer cells (Fig. 1E and F). We also observed 5hmC hypo-DMRs in many common transcriptional regulators, including *HDAC2*, *CHD8*, *E2F3*, and *KMT2A* (Supplementary Table S3). These results further support the hypothesis that 5hmC hypomethylation is a crucial regulator of gene expression in bladder cancer.

### Profiling transcriptional and methylation alternations in recurrent bladder cancer

By comparing the RNA-seq data of 31 primary and nine recurrent bladder cancer specimens using DESeq2, we identified 695 significantly upregulated genes and 197 downregulated genes (Figure S3A). Upregulated genes were mainly related to extracellular structure organization, ECM organization, angiogenesis, growth factor activity, and inflammatory responses. The downregulated genes were enriched in oxidative stress, purine catabolism, and the PPAR signaling pathway (Figure S3B). These results suggest that restructuring the ECM, inducing the generation of blood vessels, and repressing the stress response pathways are crucial for bladder cancer progression.

**Fig. 2:**
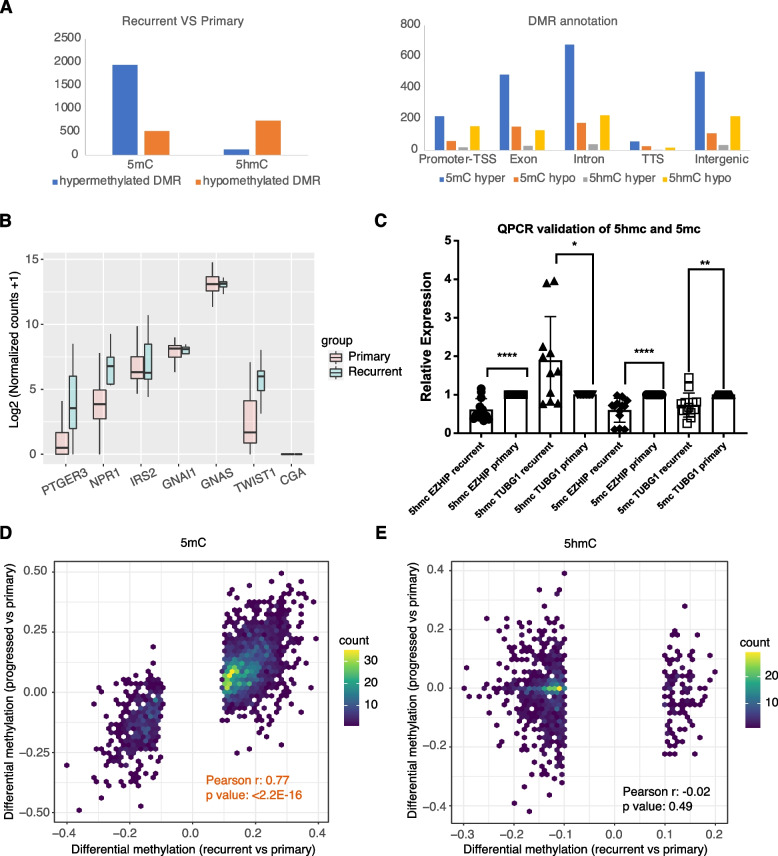
The rewiring 5mC and 5hmC landscape in recurrent bladder cancer. **A** The number of 5mC and 5hmC differentially methylated regions (DMRs) identified in recurrent bladder cancer samples (left). The genomic annotations of 5mC and 5hmC DMRs (right). **B** RNA-seq normalized count of fatty acid metabolism-related genes for 5hmC DMRs. **C** 5mC and 5hmC-specific qPCR for TUGB1 and EZHIP DMRs. **D** and **E** Correlation between differential methylation profiles in recurrent bladder cancer and five patients who developed recurrent UBC after tissue collection

Similarly, by comparing primary and recurrent bladder cancer samples, we identified 1940 5mC hyper-DMRs, 516 5mC hypo-DMRs, 122 5hmC hyper-DMRs, and 742 5hmC hypo-DMRs (Fig. 2A). Consistent with the transcriptomic alterations, 5mC hyper- and hypo-DMRs were enriched in GO terms related to EMT transition, morphogenesis of epithelial tubes, cell–cell adhesion, and mesenchymal cell proliferation. In addition, 5mC hypo-DMRs were also enriched in the WNT signaling pathway, which is usually exploited by cancer cells for prolonging survival and proliferation. Interestingly, the 5hmC hypo-DMRs were uniquely enriched in pathways related to lipolysis regulation (Figure S4).

Since targeting fatty acid metabolism inhibits the malignant phenotype of bladder cancer [29], it is worth investigating whether 5hmC can directly regulate fatty acid metabolism, which may reveal new strategies or targets for drug development. We identified 5hmC DMRs associated with fatty acid oxidation-related genes, including *TWIST1*, *CGA*, *PTGER3*, *NPR1*, *GNAS*, *IRS2*, and *GNAI1*. The 5hmC DMRs associated with *PTGER3*, *NPR1*, *GNAS*, *IRS2*, and *GNAI1* were directly annotated to promoter regions. The 5hmC DMRs associated with *TWIST1* and *CGA* were annotated to intergenic regions but overlapped with promoter-like cis-regulatory elements predicted by the ENCODE project (Figure S5). These observations suggest that 5hmC is directly involved in the regulation of the transcriptional activity of the above genes. 5hmC DMRs are hypomethylated in *TWIST1*, *NPR1*, *IRS2*, and *CGA*, and correlated negatively with their increased transcription levels. In contrast, the 5hmC DMR of *PTGER3* was hypermethylated, whereas its expression was also markedly upregulated in recurrent bladder cancers. *GNAS* and *GNAI1* were not differentially expressed in recurrent bladder cancer samples, although these were associated with the 5hmC DMR in the promoter regions (Fig. 2B). However, both genes had different transcript isoforms, suggesting that 5hmC DMRs may play a role in regulating alternative splicing (Fig. 2B and Figure S5). In summary, these results indicate that 5hmC can directly regulate the transcriptional activity of fatty acid metabolism genes in recurrent bladder cancer. Consistent with previous studies, the transcription factor to which 5hmC binds decides the mechanism by which it regulates the expression.

Since the profiling of the 5hmC level was indirect, which was calculated by subtracting oxRRBS methylation from RRBS methylation levels, we performed 5hmC-specific PCR to validate one of the most hypermethylated 5hmC DMRs that is annotated to *TUBG1,* and a top hypomethylated 5hmC DMR annotated to *EZHIP*. Consistent with the sequencing results, we observed that the 5hmC level of *TUGB1* was significantly high in recurrent bladder samples, while the 5hmC level of *EZHIP* was decreased. To examine the relationship between 5mC and 5hmC, we performed 5mC-specific PCR on these two 5hmC DMRs. In both genes, 5mC levels were decreased in recurrent bladder cancer samples, suggesting that 5hmC alternations are not always correlated negatively with 5mC alternations (Fig. 2C).

Finally, five patients were diagnosed with recurrent bladder cancer within one year after the collection of UBC tissue samples. We then compared the 5mC and 5hmC profiling of the tissue samples of these five patients with the primary bladder cancers tissues of patients who had not developed recurrent bladder cancer. Interestingly, the differential 5mC methylation of these five patients mimicked the methylation alternations observed in the recurrent bladder cancer tissues (Fig. 2D). In contrast, the differential 5hmC methylation profile of these five patients was not correlated with recurrent bladder cancer tissues (Fig. 2E). These results demonstrate that 5mC alternations related to recurrent bladder cancer occurred at an early stage during the bladder cancer progression and can be exploited as biomarkers for predicting bladder cancer prognosis. However, 5hmC alternations of recurrent bladder cancer cannot be observed in advance of the disease progression, and therefore, are less appropriate as prognostic biomarkers.

### Profiling transcriptional and methylation alternations in *PD-L1* overexpression in bladder cancer

To investigate the mechanism by which *PD-L1* is regulated in bladder cancers, we split bladder cancer samples into *PD-L1*-high and -low groups based on the median *PD-L1* expression level (Figure S6). We identified 532 significantly upregulated genes, and 23 significantly downregulated genes in the *PD-L1*-high group (Figure S7A). As expected, the upregulated genes were enriched in the negative regulation of inflammation and negative regulation of T cell activation, suggesting that *PD-L1* overexpression in bladder cancer can inhibit the immune response. The upregulated genes were the most enriched in ECM organization and cell adhesion terms. Similarly, these genes were also the most enriched in the focal adhesion-PI3K-Akt-mTOR-signaling pathway. Considering a previous study demonstrated that the focal adhesion kinase (FAK) could control *PD-L1* expression and induce immune escape, the acquisition of mobility by downregulation of focal adhesion genes is likely associated with the overexpression of *PD-L1* in bladder cancer (Figure S7B).

**Fig. 3:**
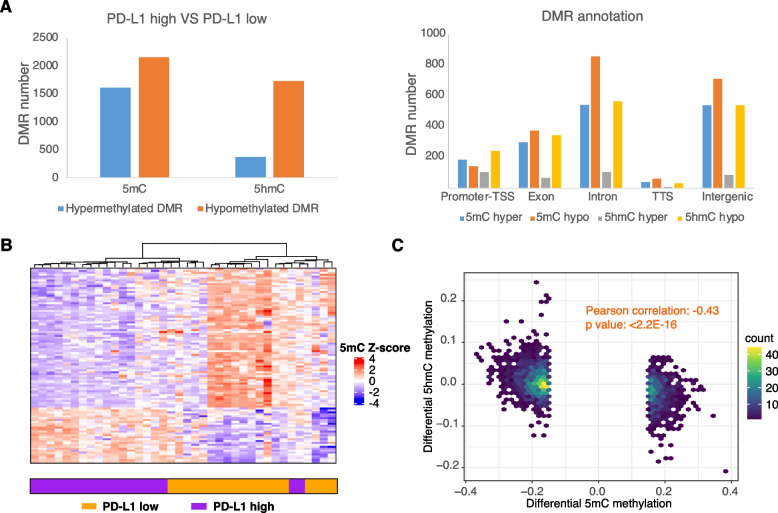
Methylation alternations in PD-L1 highly expressed bladder cancer. **A** The number of 5mC and 5hmC differentially methylated regions (DMRs) identified in PD-L1 highly expressed bladder cancer samples (left). The genomic annotations of 5mC and 5hmC DMRs (right). **B** Heatmap showing the methylation alternations in 5mC DMR biomarkers in PD-L1 highly expressed bladder cancer samples. **C** Scatter plot showing the correlation between differential 5hmC levels and differential 5mC levels in PD-L1 highly expressed bladder cancer

To investigate whether DNA methylation is involved in the immune escape process, we identified 1589 hyper 5mC DMRs, 2149 hypo 5mC DMRs, 370 hyper 5hmC DMRs, and 1725 hypo 5hmC DMRs in samples with high PD-L1 expression levels (Fig. 3A). The hyper 5mC DMRs were highly enriched in the maturity-onset diabetes of the young, basal cell carcinoma, and Hippo signaling pathways. In contrast, hypo 5mC DMRs were highly enriched in adherens junction assembly and negative regulation of EMT transition, and positive regulation of the apoptotic signaling pathway (Figure S8). Among the 532 upregulated genes in PD-L1 highly expressed samples, 49 genes corresponded to 5mC hypo DMRs. These genes, including *CCL11*, *CCL20*, and *S1PR1*, were significantly enriched in the cellular response to cytokine stimuli and T-cell migration (Figure S9A).

The hyper 5hmC DMRs were enriched in the bladder cancer pathway, toll-like receptor signaling, and stress-activated MAPK cascade. In contrast, the hypo 5hmC DMRs were enriched in the insulin signaling pathway, regulation of lipolysis in adipocytes, focal adhesion, and the AMPK signaling pathway (Figure S8B). Among the transcriptionally upregulated genes in samples with highly expressed *PD-L1* levels, 51 genes were associated with 5hmC hypo DMRs. These genes were enriched in ECM organization and negatively regulated the inflammatory response to antigenic stimuli (Figure S9B).

### DNA methylation biomarkers for predicting immunotherapy responses

Here, we compared the RRBS-seq and oxRRBS-seq data between *PD-L1*-high and -low bladder cancer samples and identified methylation markers with optimal performance in predicting samples with a highly expressed *PD-L1*. By univariate analysis, we identified 102 5mC DMR biomarkers with significant methylation differences and predicted an area under the curve (AUC) score of more than 0.8 (Fig. 3B). In contrast, there were only 21 5hmC DMRs with AUC scores higher than 0.8, and their methylation difference was less significant than that of 5mC DMRs, indicating that 5mC biomarkers are more appropriate for predicting high *PD-L1* expression in bladder samples.

**Fig. 4:**
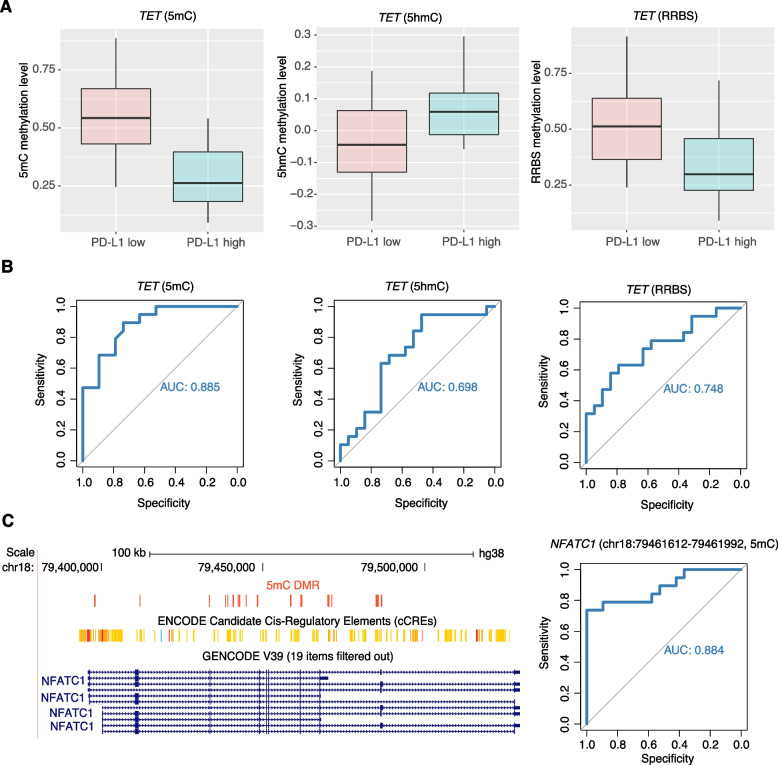
Utilizing 5mC biomarkers for predicting immunotherapy responses. **A** 5mC, 5hmC, and RRBS methylation level alternations in the *TET* gene. **B** The area under the curve (AUC) score for 5mC, 5hmC, and RRBS methylation in *TET* for predicting PD-L1 highly expressed bladder cancer. **C** Track plot showing the 5mC DMRs annotated to NFATC1 (left). The AUC score of one NFATC1 DMR for predicting PD-L1 highly expressed bladder cancer (right)

In most cell-free DNA methylation biomarker studies, the methylation level is profiled using bisulfite-based or similar enzymatic technologies that convert both 5mC and 5hmC into uracil, and therefore, cannot distinguish between 5mC and 5hmC signals. Because 5hmC is a product of the active demethylation process of 5mC, 5hmC methylation alterations are globally anti-correlated with 5mC methylation alterations, which was indeed observed when comparing *PD-L1*-high and *PD-LI*-low samples (Fig. 3C). Given that 5hmC has a worse predicted AUC score and undergoes opposite methylation alterations than 5mC biomarkers, the oxRRBS-seq, which only profiles 5mC, can be used to identify better markers than the commonly used RRBS-seq. As proof of principle, we examined one of the top-performing DMR markers annotated to the *TET* gene. This DMR is 5mC-hypomethylated, whereas 5hmC is hypermethylated in *PD-L1*-high bladder cancer samples. When the methylation alternation was profiled using RRBS, this *TET* DMR was found to be hypomethylated but the methylation difference was less significant than the 5mC alternation (Fig. 4A). Correspondingly, the 5mC level of this *TET* DMR had the highest predicted AUC score compared to those of the 5hmC and RRBS methylation levels (Fig. 4B).

Notably, among the 102 identified 5mC DMR biomarkers, 5 DMRs were annotated to the *NFATC1* gene, which plays a crucial role in T cell activation and is involved in the regulation of PD-1/PD-L1 signaling. By lowering the AUC score cut-off, 16 significant 5mC DMRs were identified in the *NFATC1* gene body. Most of these DMRs were hypomethylated and co-localized with the *NFATC1* exons, and encoded cis-regulatory element regions, suggesting that these 5mC alterations were involved in the transcriptional regulation of *NFATC1*. The best-performing DMR (chr18:79,461,612–79,461,992), which is close to the seventh exon of *NFATC1*, had an AUC score of 0.884 for *PD-L1*-high samples (Fig. 4C). Altogether, we demonstrated the potential of using DNA methylation markers for predicting immunotherapy responses in patients with bladder cancer and the advantages of using oxRRBS-seq for discovering methylation markers.

## Discussion

To comprehensively investigate the development of recurrent bladder cancer and the PD-L1 overexpression resulting in tumor immune escape, we performed multi-omics experiments to delineate genetic, 5mC, and 5hmC alternations during the above processes. By WES, we identified mutations in many known UBC driver genes, including *KDM6A, TP53,* and *FGFR3*. However, few of these driver mutations are enriched in recurrent bladder cancer or PD-L1 over-expressing cases (Figure S10). In contrast, significant 5mC and 5hmC DMRs were identified in various pathways that are associated with cancer progression and immune response. These results indicate that epigenetic alternations are highly involved in the progression of UBC.

The hydroxymethylation level mediated by TET proteins could help regulate fatty acid metabolism. TET1 is recruited by peroxisome proliferator-activated receptors α and γ (PPARα and PPARγ) to induce the demethylation of their response element region and interfere with fatty acid metabolism [30, 31]. A decrease in TET1 leads to the upregulation of genes involved in lipogenesis and fatty acid uptake, as well as the downregulation of genes related to lipolysis and fatty acid oxidation [31]. Notably, elevated lipogenesis is predictive of poor prognosis in certain tumor types [32]. Previous studies on the metabolic characteristics of cancer cells have demonstrated that, unlike normal cells, cancer cells require larger amounts of fatty acids because of the higher demand for the synthesis of signaling molecules, cellular structural elements, and adenosine triphosphate [33, 34]. The fatty acid synthase (FASN) expression in bladder cancer is significantly upregulated and is regarded as an adverse prognostic factor for the recurrence and progression of bladder cancer [35–37]. Tao et al. found that a small interfering RNA of FASN could upregulate E-cadherin expression and downregulate Snail expression in bladder cancer cells, suggesting that recurrent bladder cancer may be associated with FASN-induced EMT [38]. In this study, we specifically identified that fatty acid oxidation-related genes were significantly associated with 5hmC-induced transcription alteration in recurrent bladder cancers. These results enrich the current knowledge of how 5hmC contributes to metabolism regulation through epigenetic regulation.

Escaping immune surveillance is a hallmark of cancer progression. One way of escaping the immune system is through *PD-L1* overexpression by cancer cells, which binds to PD1 on the immune cell surface and inactivates the cancer cell immune response. Given its promising anti-tumor effect, the PD1/PD-L1 inhibitor (pembrolizumab) has been approved to treat bladder cancers. However, less than half of bladder cancer patients respond to immunotherapy. *PD-L1* expression has been well established as a biomarker for identifying patients who are more likely to benefit from immunotherapy. Advanced technologies for cell-free DNA methylation detection further make it possible to develop non-invasive blood tests to pre-select immunotherapy-responsive patients. According to our results, hypomethylation of the NFATc1 gene body was observed in bladder cancer cells, indicating an activating state of NFATc1 transcription, and this state could be used to predict PD-L1 expression. NFATc1 is one of the main isoforms of NFAT expressed in T cells and plays an essential role in regulating gene transcription in response to T-cell receptor (TCR)-mediated signals [39–41]. NFATc1 can also be activated by the B cell receptor (BCR) and upregulate the IL-10 chemokine to activate the JAK2/STAT3 pathway in B cell lymphoma cells, ultimately inducing PD-L1 expression [42]. The correlation between NFATc1 and PD-L1 expression is similar in bladder cancer. According to Kawahara et al., PD-L1 is more highly expressed in high-grade bladder cancer than in low-grade bladder cancer and correlates positively with the expression of NFATc1 genes [43]. Therefore, the STAT1-PD-L1-NFATc1 pathway was proposed to reveal the immunosuppressive mechanism of PD-L1, which may increase the potential for PD-L1-based antitumor immunotherapy for bladder cancer [43]. In this study, we built a machine learning-based model to directly predict PD-L1-high bladder cancers based on the methylation level of NFATc1 and achieved an AUC score of 0.884. Future studies should focus on whether this precise prediction can be achieved using cell-free DNA.

In the present study, we employed improved methods of genome-wide methylation and hydroxymethylation mapping by combining oxRRBS with traditional RRBS. Standard RRBS cannot distinguish between 5mC and 5hmC, thus rendering the summation of 5mC and 5hmC levels in previous studies. In oxRRBS, selective oxidation of 5hmC to fC can be achieved using potassium perruthenate. The hydroxymethylation level can be determined by subtracting the bisulfite sequencing signals obtained from oxRRBS from those of RRBS [28, 44, 45]. Therefore, an accurate 5mC and 5hmC fraction could be separately calculated using RRBS + oxRRBS, thus providing more detailed epigenetic information at a higher resolution. This is more suitable as a biomarker for bladder cancer diagnosis and prognosis.

## Supplementary Information

Additional file 1: Figure S1. Mutation Analysis of the bladder cancer cohort.Oncoplot of the 44 UBC samples..Top 10 mutated genes in the UBC cohorts.. Top 10 pathways that were affected by the mutations in this UBC cohort.. Co-barplot with TCGA bladder cancer dataset showed the mutation frequency of the driver genes of the bladder cancers.. Variant allele frequency of the driver genes of the bladder cancer samples. Numbers in the first line represent the mutation hits in each gene in this cohort. Figure S2. Differential expression analysis between bladder cancer and paracancerous samples.Volcano plot for differentially expressed genes in bladder cancer samples.Pathway enrichment analysis of up-regulated and down-regulated genes in bladder cancer samples. Figure S3. Differential expression analysis between recurrent and primary bladder cancer samples.Volcano plot for differentially expressed genes in recurrent bladder cancer samples.Pathway enrichment analysis of up-regulated and down-regulated genes in recurrent bladder cancer samples. Figure S4. Pathway enrichment of 5mC and 5hmC DMRs in recurrent bladder cancer samples. Figure S5. Track plots of 5hmC DMRs annotated to fatty acid metabolism genes in recurrent bladder cancer samples. Figure S6. PD-L1 expression level in PD-L1 high and PD-L1 low UBC samples. Figure S7. Differential expression analysis between PD-L1-high and -low bladder cancer samples.Volcano plot for differentially expressed genes in PD-L1-high bladder cancer samples.Pathway enrichment analysis of up-regulated in PD-L1-high bladder cancer samples. Figure S8. Pathway enrichment of 5mC and 5hmC DMRs in PD-L1-high bladder cancer samples. Figure S9. Pathway enrichment of 5mC hypo DMRs and 5hmC hypo DMRs that are associated with significant DEGs in PD-L1 highly expressed bladder cancer. Figure S10. Driver mutations in recurrent and PD-L1 overexpression UBC samples.Please check if the additional files are captured and presented correctly.We confirmed that additional files are captured and presented correctly.As per journal requirements, every additional file must have a corresponding caption. In this regard, please be informed that the caption was taken from the additional e-file itself. Please advise if the action taken is appropriate and amend if necessary.We confirmed that this action is correct and no futher amend is necessary.

Additional file 2: Table S1. Differentially expressed genes in bladder cancers, recurrent bladder cancers and PD-L1 highly expressed bladder cancers.

Additional file 3: Table S2. Significant 5mC differentially methylated regions in bladder cancers, recurrent bladder cancers and PD-L1 highly expressed bladder cancers.

Additional file 4: Table S3. Significant 5hmC differentially methylated regions in bladder cancers, recurrent bladder cancers and PD-L1 highly expressed bladder cancers.

## Data Availability

All sequencing data can be accessed through the following link: https://www.biosino.org/node/project/detail/OEP004094.
