# Supplementary material for: Integrative multi-omics analysis depicts the methylome and hydroxymethylome in recurrent bladder cancers and identifies biomarkers for predicting PD-L1 expression
Source: Biomark Res. 2023 May 3;11:47. doi: 10.1186/s40364-023-00488-3 (PMC10155358; doi:10.1186/s40364-023-00488-3)
Supplement: Supplementary file 1 — Additional file 1: Figure S1. Mutation Analysis of the bladder cancer cohort.Oncoplot of the 44 UBC samples..Top 10 mutated genes in the UBC cohorts.. Top 10 pathways that were affected by the mutations in this UBC cohort.. Co-barplot with TCGA bladder cancer dataset showed the mutation frequency of the driver genes of the bladder cancers.. Variant allele frequency of the driver genes of the bladder cancer samples. Numbers in the first line represent the mutation hits in each gene in this cohort. Figure S2. Differential expression analysis between bladder cancer and paracancerous samples.Volcano plot for differentially expressed genes in bladder cancer samples.Pathway enrichment analysis of up-regulated and down-regulated genes in bladder cancer samples. Figure S3. Differential expression analysis between recurrent and primary bladder cancer samples.Volcano plot for differentially expressed genes in recurrent bladder cancer samples.Pathway enrichment analysis of up-regulated and down-regulated genes in recurrent bladder cancer samples. Figure S4. Pathway enrichment of 5mC and 5hmC DMRs in recurrent bladder cancer samples. Figure S5. Track plots of 5hmC DMRs annotated to fatty acid metabolism genes in recurrent bladder cancer samples. Figure S6. PD-L1 expression level in PD-L1 high and PD-L1 low UBC samples. Figure S7. Differential expression analysis between PD-L1-high and -low bladder cancer samples.Volcano plot for differentially expressed genes in PD-L1-high bladder cancer samples.Pathway enrichment analysis of up-regulated in PD-L1-high bladder cancer samples. Figure S8. Pathway enrichment of 5mC and 5hmC DMRs in PD-L1-high bladder cancer samples. Figure S9. Pathway enrichment of 5mC hypo DMRs and 5hmC hypo DMRs that are associated with significant DEGs in PD-L1 highly expressed bladder cancer. Figure S10. Driver mutations in recurrent and PD-L1 overexpression UBC samples.Please check if the additional files are captured and presented correctly.We confirmed that [file 40364_2023_488_MOESM1_ESM.docx]

**Supplementary Figures**


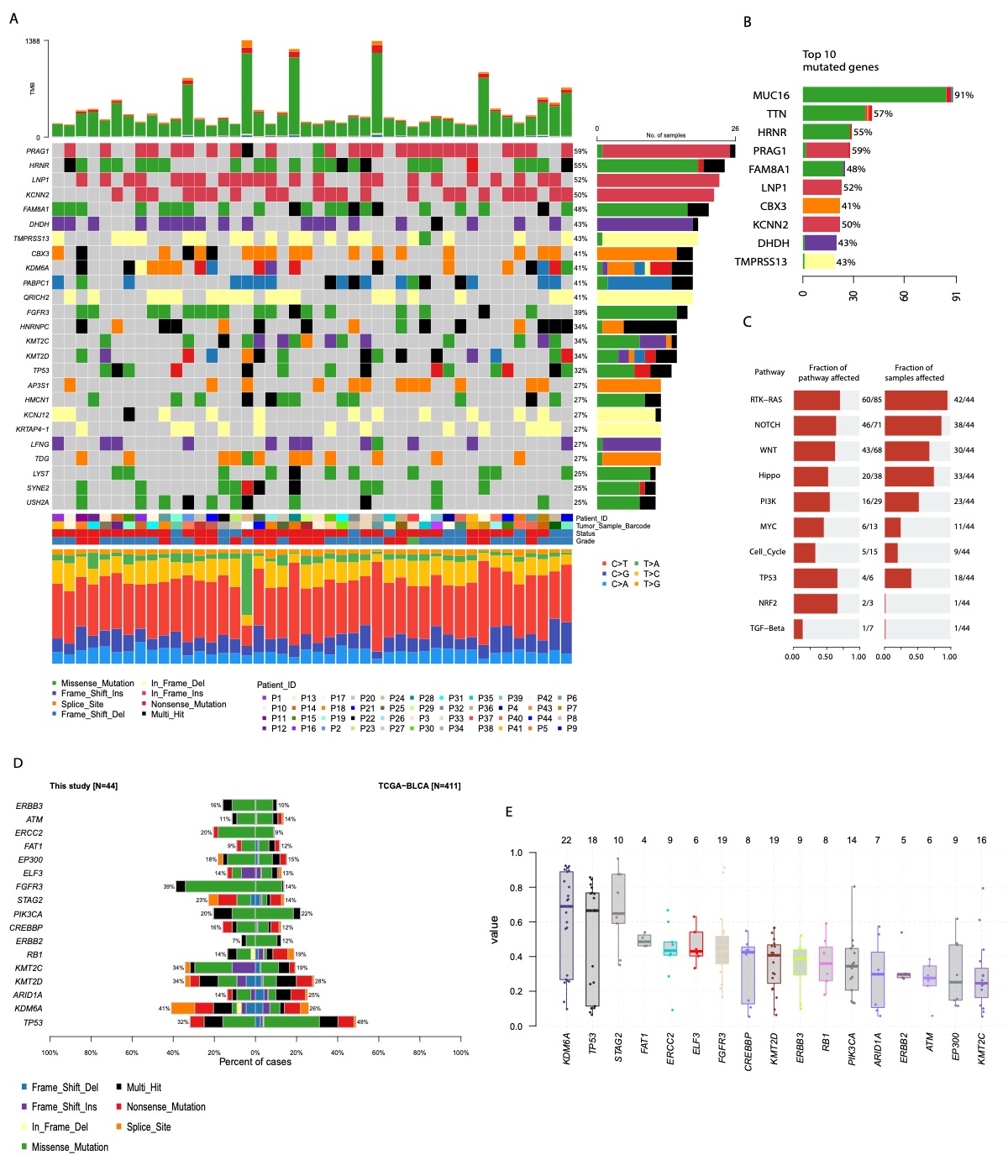


**Figure S1. Mutation Analysis of the bladder cancer cohort.** (A) Oncoplot of the 44 UBC samples. (blue: recurrent, high-grade, red: primary, low-grade). (B) Top 10 mutated genes in the UBC cohorts. (C). Top 10 pathways that were affected by the mutations in this UBC cohort. (D). Co-barplot with TCGA bladder cancer dataset showed the mutation frequency of the driver genes of the bladder cancers. (E). Variant allele frequency of the driver genes of the bladder cancer samples. Numbers in the first line represent the mutation hits in each gene in this cohort.


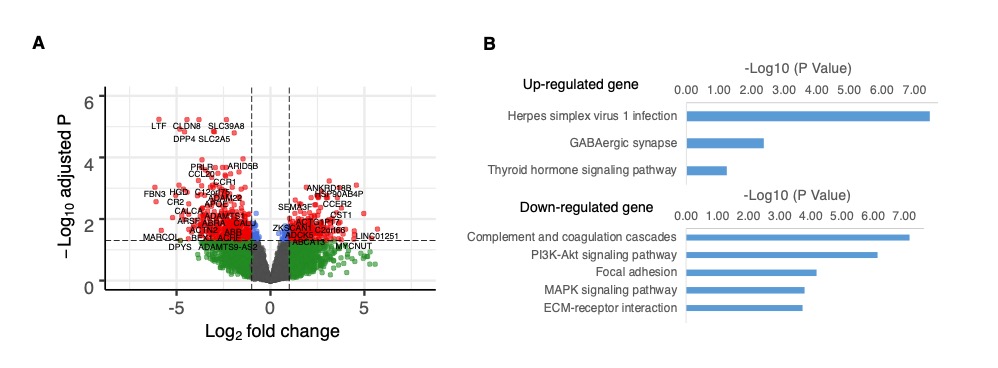


**Figure S2. Differential expression analysis between bladder cancer and paracancerous samples.** (A) Volcano plot for differentially expressed genes in bladder cancer samples. (B) Pathway enrichment analysis of up-regulated and down-regulated genes in bladder cancer samples.


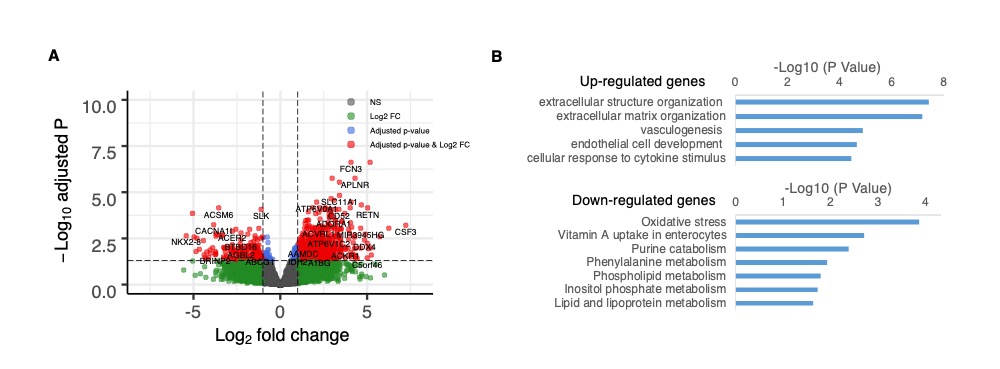


**Figure S3. Differential expression analysis between recurrent and primary bladder cancer samples.** (A) Volcano plot for differentially expressed genes in recurrent bladder cancer samples. (B) Pathway enrichment analysis of up-regulated and down-regulated genes in recurrent bladder cancer samples.

**Figure S4. Pathway enrichment of 5mC and 5hmC DMRs in recurrent bladder cancer samples.**

**Figure S5. Track plots of 5hmC DMRs annotated to fatty acid metabolism genes in recurrent bladder cancer samples.**


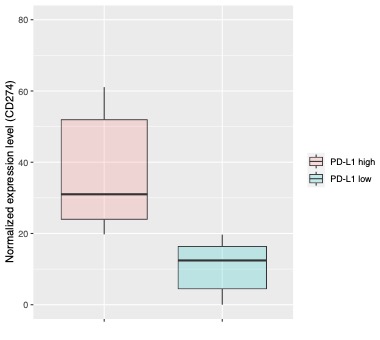


**Figure S6. PD-L1 expression level in PD-L1 high and PD-L1 low UBC samples**


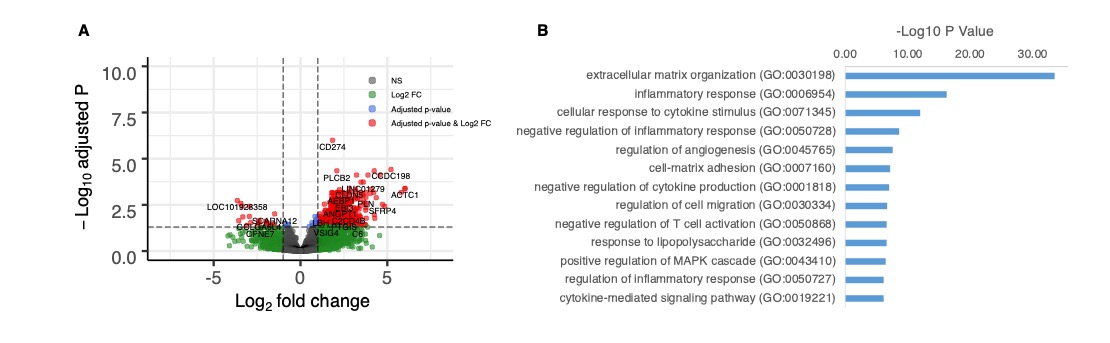


**Figure S7. Differential expression analysis between PD-L1-high and -low bladder cancer samples.** (A) Volcano plot for differentially expressed genes in PD-L1-high bladder cancer samples. (B) Pathway enrichment analysis of up-regulated in PD-L1-high bladder cancer samples.

**Figure S8. Pathway enrichment of 5mC and 5hmC DMRs in PD-L1-high bladder cancer samples.**

**Figure S9. Pathway enrichment of 5mC hypo DMRs and 5hmC hypo DMRs that are associated with significant DEGs in PD-L1 highly expressed bladder cancer.**

**Figure S10. Driver mutations in recurrent and PD-L1 overexpression UBC samples.**
